# Identification of *wysPII* as an Activator of Morphological Development in *Streptomyces albulus* CK-15

**DOI:** 10.3389/fmicb.2018.02550

**Published:** 2018-10-25

**Authors:** Binghua Liu, Beibei Ge, Jinjin Ma, Qiuhe Wei, Abid Ali Khan, Liming Shi, Kecheng Zhang

**Affiliations:** ^1^Institute of Plant Protection, Chinese Academy of Agricultural Sciences, Beijing, China; ^2^Centre of Biotechnology and Microbiology, University of Peshawar, Peshawar, Pakistan

**Keywords:** *Streptomyces albulus*, *wysPII*, regulatory genes, LuxR family, morphological development

## Abstract

Wuyiencin is produced by *Streptomyces albulus* var. *wuyiensis* and used widely in agriculture to control a variety of fungal diseases, such as cucumber downy mildew, strawberry powdery mildew, and tomato gray mold. As an industrially-produced biopesticide, reducing production costs is very important for popularization of this approach. To obtain a rapidly growing strain that effectively shortens the fermentation time, we investigated the effects of knockout and overexpression of the *wysPII* gene, a member of the LuxR regulatory gene family, in *S. albulus* strain CK-15. The Δ*wysPII* mutant exhibited a reduced rate of growth and sporulation. The time taken to reach the greatest mycelial biomass was approximately 18 h shorter in the ooPII (*wysPII* overexpressing) strain compared with that of the wild-type (WT) strain. In addition, the time to reach the greatest wuyiencin production was 56 h in the ooPII strain compared with 62 h in the WT strain. Furthermore, *wysPII* was shown to act as an activator of morphological development without affecting wuyiencin production. Thus, the ooPII strain can be used to reduce costs and increase efficiency in industrial fermentation processes for wuyiencin production.

## Introduction

*Streptomyces* are soil-dwelling, Gram-positive, filamentous bacteria, with a complex life cycle that involves formation of substrate mycelium, aerial mycelium, and spores (McCormick and Flardh, 2011). Many members of this bacterial lineage produce secondary metabolites including a variety of antibiotics (Bibb, 2005). Production of secondary metabolites is tightly regulated by complicated transcriptional processes involving multiple levels of regulation (Martín, 2004; Liu et al., 2013). Pathway-specific transcriptional regulation, which is the most fundamental level, is mediated by regulatory genes encoded within the respective biosynthetic gene clusters (Vicente et al., 2015). Some regulatory factors affect antibiotic production, while others affect morphological development and production of antibiotics via processes that are sometimes genetically coupled by shared transcriptional regulators (Hur et al., 2008; Ge et al., 2016). A few regulatory factors are involved only in morphological development, an example being MtrA, which is crucial for normal development of aerial hyphae of the model strain *S. coelicolor* (Cui et al., 2016; Zhang et al., 2017).

The LuxR family of regulators plays important roles in acyl-homoserine lactone-mediated quorum sensing in Gram-negative bacteria (Nasser and Sylvie, 2007), regulating many physiological processes, such as bioluminescence (Antunes et al., 2008), plasmid transfer (Danino et al., 2003), biofilm formation (Alonso-Hearn et al., 2010), as well as production of virulence factors (Passador et al., 1993), extracellular enzymes (Andersson et al., 2000), and secondary metabolites (Latifi et al., 1995). The LuxR superfamily comprises transcriptional regulators with a DNA-binding helix-turn-helix (HTH) motif in the C- and the N-terminal regions, and, frequently, an autoinducer-binding or response regulatory domain (De and Raaijmakers, 2009). HTH domains of approximately 65 amino acids are present in LuxR family transcriptional regulators. Most LuxR-type regulators activate transcription, but some function as repressors or have a dual role at different sites. These regulators control a large number of activities. LuxR-type HTH proteins can be activated by a two-component sensory transduction system in which the protein is activated by phosphorylation, normally on an aspartate residue (e.g., FixJ of *Sinorhizobium meliloti*) (Birck et al., 2002), or by binding to *N*-acyl-homoserine lactones (e.g., LuxR of *Vibrio fischeri*) (Fuqua et al., 1996). The LuxR/FixJ family also contains autonomous effector domain regulators, represented by GerE, which regulates spore formation in *Bacillus subtilis* (Ducros et al., 2001) and multiple ligand-binding regulators, exemplified by MalT in *Escherichia coli*, which functions as a maltose regulator (Schlegel et al., 2002). Most LuxR family members are identified as activators of antibiotic production and morphological development (Yu et al., 2012). LuxR family members include PimM, which positively regulates pimaricin biosynthesis in *Streptomyces natalensis* (Antón et al., 2007), NysRI, NysRII, and NysRIII, which regulate the *S. noursei* ATCC11455 nystatin biosynthetic pathway (Sekurova et al., 2004), TmcN, which is present in the tautomycetin biosynthetic pathway of *Streptomyces* sp. CK4412 (Hur et al., 2008), and GdmRI and GdmRII, which regulate the *S. hygroscopicus* 17997 geldanamycin biosynthetic pathway (He et al., 2008).

Wuyiencin is a nucleoside antibiotic produced by *S. ahygroscopicus* var. *wuyiensis*, first isolated from soil in the Wuyi Mountains, China (Wei et al., 1984). This strain was previously designated as *S. ahygroscopicus*, depending on presence or absence of hygroscopicity; however, based on further molecular identification and other characteristics, *S. ahygroscopicus* CK-15 was renamed *S. albulus.* As an efficient, broad-spectrum biological fungicide, wuyiencin is used in agriculture to control a variety of fungal diseases, such as cucumber downy mildew, strawberry powdery mildew, and tomato gray mold, with high efficiency and low toxicity compared with chemical pesticides (Zeng et al., 1996; Sun et al., 2003; Ge et al., 2015a). Wuyiencin meets national standards for organic food production (COFFC-R-0903-0070). Previously, we sequenced the genome of *S. albulus* var. *wuyiensis* strain CK-15 and analyzed the wuyiencin biosynthetic cluster (Ge et al., 2014, 2015b). WysR, a member of the PAS-LuxR family of regulators and present in this gene cluster, was identified as a transcriptional activator of wuyiencin biosynthesis. Overexpression of *wysR* increased the production of wuyiencin by almost threefold compared with the wild-type (WT) strain (Liu et al., 2014). WysR3, a novel member of the DeoR family of regulators, had no effect on antibiotic production, but acted as a repressor during morphological development. The Δ*wysR3* strain grew faster than the WT, reaching the plateau stage of maximum biomass 12 h earlier in the flasks (Ge et al., 2016).

In this study, we characterized WysPII, a LuxR family protein encoded within the wuyiencin biosynthesis gene cluster. *WysPII* was shown to act as an activator during morphological development of *S. albulus* CK-15. Furthermore, a *wysPII* overexpression strain (ooPII) exhibited much more rapid wuyiencin production (shorter time to reach maximum) and increased growth compared with the WT strain. The ooPII strain is of significant industrial utility due to its potential to shorten the production process of wuyiencin by effectively reducing the time required for fermentation. These properties will promote the industrial application of wuyiencin.

## Materials and Methods

### Bacterial Strains, Plasmids, and Growth Conditions

Table 1 lists bacterial strains and plasmids used in this work. *S. albulus* var. *wuyiensis* strain CK-15 (China General Microbiological Culture Collection Center No. 0703; hereafter called *S. wuyiensis* CK-15) was cultured at 28°C on mannitol-soybean (MS) agar or in yeast extract malt extract (YEME) liquid medium (Hobbs et al., 1989). Fermentation medium contained (per 100 ml) 2 g soybean flour, 2 g glucose, 3 g corn starch, 300 mg CaCO_3_, and 400 mg (NH_4_)_2_SO_4_. *E. coli* DH5α was used for DNA manipulation and was grown in Luria–Bertani (LB) liquid broth or on LB-agar. The vector pEASY-T1-Simple was used for general cloning, pKC1139 was used as a disruption vector, and pSETC (Labes et al., 1997) for complementation and overexpression by standard procedures (Kieser, 2000; Liu et al., 2014). pSETC contains the constitutive and strong expression promoter P_SF14_ incorporated into the genome-integrating plasmid pSET152. Non-methylating *E. coli* strain ET12567/pUZ8002 was used for gene conjugation from *E. coli* to *S. albulus* CK-15. *Rhodotorula rubra* N-1 was used as the indicator strain in disk-agar wuyiencin diffusion assays. Apramycin (50 μg/ml) was used in LB medium as required.

**Table 1:** Strains and plasmids used in this study.

Strains and plasmids	Description
pEASY-T1 Simple (pT1)	Cloning vector
pT1-A	pEASY-T1-containing fragment of an upstream region of *wysPII*
pT1-B	pEASY-T1-containing fragment of a downstream region of *wysPII*
*E. coli* DH*5α* strain	A host for plasmid cloning
pKC1139	Plasmid used for gene-knockout in *Streptomyces*
pKC1139-Δ*wysPII*	*wysPII* disruption plasmid based on pKC1139
pSETC	Plasmid used for gene complementation
pSETC- *wysPII*	*wysPII* overexpression and complementation plasmid based on pSETC
*S. albulus* CK-15	Wild-type wuyiencin producer
*E. coli ET12567/pUZ8002*	Methylation-deficient *E. coli* for conjugation with the helper plasmid
*Rhodotorula rubra* N-1	Indicator strain for wuyiencin bioassays

### DNA Manipulation and Sequence Analysis

Using a *Streptomyces* DNA Isolation Kit (GenMed Scientifics Inc., United States), total *S. albulus* CK-15 genomic DNA was isolated from mycelia after 72 h of cultivation on solid MS medium supplemented with 0.3% malt extract, 0.3% yeast extract, 0.5% tryptone, and 4% glucose, or from mycelia after 48 h of culture in YEME liquid medium. PCR amplification used 2×*Taq* PCR Mix (Bingda, China). The amplification conditions were: 95°C for 5 min; 32 cycles of 95°C for 30 s, 59°C for 30 s, and 72°C for 2 min; and a final extension at 72°C for 10 min. Primers were designed using Primer Premier 5 and the DNA sequences were sequenced by BGI Tech (Beijing, China). DNAMAN software was used to compare nucleotide and protein sequences.

### *WysPII* Deletion Mutant

A *wysPII* deletion mutant was generated by homologous recombination. Primers AF and AR were used to amplify the upstream region (1,504 bp) of *wysPII* (Table 2) and BF and BR to amplify the downstream region (1,357 bp). The resulting DNA fragments were ligated into pEASY-T1-Simple and digested with *Hind*III/*Xba*I or *Xba*I/*Eco*RI to yield plasmids pT1-A and pT1-B, respectively. The upstream and downstream region sequences were then ligated into pKC1139 to generate the disruption plasmid pKC1139-Δ*wysPII*, which was then transferred into *S. albulus* CK-15 by conjugation. Plates were incubated at 28°C for 16 h, then overlaid with sterile water (1 ml) containing 50 μg/ml apramycin and 25 μg/ml nalidixic acid. The plates were further incubated at 34°C until conjugants appeared. Double-crossover recombinants were selected on the basis of apramycin sensitivity followed by confirmation by PCR using primers TF and TR (Table 2).

**Table 2:** Primers used in this study.

Primer	Sequence (5′–3′)	Description
AF	AAGCTTATACGGCTTCACCTGCCTCGC	Upstream region of
AR	TCTAGACCGCTACCTCTCGTCCTGTTCG	*wysPII*
BF	TCTAGACTGAGCAACGCCTACCGCAAG	Downstream region of
BR	GAATTCGCTGTCGTCCTGGGTGCGGT	*wysPII*
TF	CCAGCGTCTACCGGAAGCTC	Identification of
TR	GGCGATGCCGAAAGGGAAGT	Δ*wysPII*
Am-F	GAGTGCAATGTCGTGCAATACGA	Identification of the
Am-R	GCATTCTTCGCATCCCGCCT	Am-resistance gene
Com-F	TCTAGAGAACAGGACGAGAGGTAGCG	Complementation of
Com-R	GAATTCCCAGTTCGTTCTCCCGCTC	*wysPII*
*bldA*-F	CGGTGACGGGTGGACAGG	*bldA* gene fragment
*bldA*-R	TCCATCGCCAACACCCCA	
*bldB*-F	CCGCAGCCGACAAGGAAT	*bldB* gene fragment
*bldB*-R	CCTCGGTGTAGCGCAGCA	
*whiA*-F	AACCGCCTCGCCAACTTC	*whiA* gene fragment
*whiA*-R	ACGCCTGCTTGTGCTCCAT	
*whiB*-F	CGACCCCGAGTCCTTCTTC	*whiB* gene fragment
*whiB*-R	GTCGTTGGCGAGGGCAT	
*whiH*-F	CATCGAGTGGCGTGCCTAC	*whiH* gene fragment
*whiH*-R	TGGAGGAGCAGGGTGTGGA	
*whiI*-F	CCGTCACCCGCCATCTGT	*whiI* gene fragment
*whiI*-R	CCAGCCGCAAGACCTCC	
*ramA*-F	AGTGGCGGCGCTGTTCA	*ramA* gene fragment
*ramA*-R	GCCTGGGCCTGCTGGTAA	
*ramB*-F	GACTCCTGGTGGTGCTCGAA	*ramB* gene fragment
*ramB*-R	GGTGCCGTAGGAGAAGGTGG	
*ramC*-F	TCTCAGCCTGCCTGGACAAC	*ramC* gene fragment
*ramC*-R	GCGGGGTAGACGGTCAGG	
*ramR*-F	GCTCAACACCGCCGACATT	*ramR* gene fragment
*ramR*-R	ACCGTGCCCTTGCTCAGATA	
*hrdB*-F	CCGAGTCTGTGATGGCGCT	*hrdB* gene fragment
*hrdB*-R	CTCGTCGAGGATTTGGTTGA	

### Complementation of the *wysPII* Mutant

For complementation of *wysPII* disruptants, *wysPII* was amplified using the genomic DNA template with primers Com-F and Com-R. The fragment was digested with *Hin*dIII and *Eco*RI, and the product (2,856 bp) was ligated into the *Hin*dIII/*Eco*RI sites of pSETC to yield pSETC-*wysPII*. This plasmid was first transformed into *E. coli* ET12567 and then transferred into *S. albulus* CK-15 Δ*wysPII* by intergeneric conjugation to yield strain *S. wuyiensis* comPII. Apramycin-resistant transconjugants were confirmed by PCR. The phenotype of the resulting complementation strain was assessed by apramycin selection (50 μg/ml) on MYM medium.

### Overexpression of *wysPII*

pSTEC-*wysPII* was introduced into WT *S. albulus* CK-15 by conjugation to yield the *S. albulus* ooPII strain. The empty vector pSETC was also transferred into WT *S. albulus* CK-15 as a control. Apramycin-resistant transconjugants were confirmed by PCR using primers Am-F and Am-R, and the phenotype of the resulting overexpression strain was determined as above.

### Wuyiencin Production Assay

Fresh mature seed solution was inoculated with spores (1.0 × 10^6^) in 50 ml YEME medium and incubated at 28°C for 20 h. Seed cultures were transferred into 100 ml 10% fermentation medium and incubated at 28°C with agitation (220 rpm) for 64 h. The fermentation broth was filtered using Millipore express membrane filters (pore diameter 0.22 μm; Merck kGaA Darmstadt, Germany). The antibacterial effects of the broth against *R. rubra* were tested by measuring the diameter of bacteriostasis circles.

For the actual wuyiencin production time-course analysis, fermentation broth was collected by filtration using Millipore express membrane filters (pore diameter 0.22 μm; Merck) at 2-h intervals for 72 h. Wuyiencin concentrations in the broth were also determined using an Agilent 1100 high-performance liquid chromatography (HPLC) system with an SB-AQ column (4.6 mm × 250 mm i.d., 5 μm) at 25°C. The mobile phase was 1.4 g/L C_2_HCl_3_O_2_ (flow rate 1.5 ml/min) with detection at 254 nm. The wuyiencin standard was prepared using the following series of purification steps: oxalic acid precipitation, macroporous resin HP-20 adsorption, HW-40C and HW-40F resin adsorption and lyophilization. Wuyiencin had a retention time of 15 min in these conditions.

### Measurement of Mycelial Biomass

Fresh mature seed solution was inoculated into 50 ml M3G medium [10 g (NH4)_2_SO_4_, 50 g glucose, 5.0 g yeast powder, 1.36 g KH_2_PO_4_, 0.8 g K_2_PO_4_⋅3H_2_O, 0.5 g MgSO_4_⋅7H_2_O, 0.04 g ZnSO_4_⋅7H_2_O, and 0.03 g FeSO_4_⋅7H_2_O, per liter, pH 7.0] and incubated at 28°C with agitation at 220 rpm for 48 h to produce a seed culture. The seed culture for each strain was then inoculated at 2% into 100 ml M3G medium and incubated at 28°C (220 rpm). Samples were collected every 6 h and dried to constant weight at 90°C on filter papers. The difference in weight of the paper before and after filtration represented the dry weight of mycelium in the fermented liquid sample. The strains (WT, Δ*wysPII*, comPII and ooPII) were also cultured at 28°C on MS, ISP 2 (4 g glucose, 10 g malt infusion, 4 g yeast extract, 17 g agar, per liter, pH 7.0–7.4) and ISP 3(20 g oats, 0.5 g K_2_HPO_4_, 0.2 g KNO_3_, 0.2 g MgSO_4_, 17 g agar, per liter) media to evaluate differences in the mycelial biomass of these strains at specific time-points.

### Isolation of Total RNA and Reverse Transcription-Quantitative PCR

The WT strain and ooPII strains were grown at 28°C on MS medium plate for 72 h. The sample was first ground in a pre-cooled mortar, frozen in liquid nitrogen and powdered before Trizol Reagent (CWBIO) was added. The Ultrapure RNA Kit (CWBIO) was used to isolate total RNA according to the manufacturer’s instructions. The crude RNA samples were treated with the DNase I (TIANGEN) to degrade the chromosomal DNA.

cDNA reverse transcription was performed using the PrimeScript™ RT reagent Kit(TaKaRa) with gDNA Eraser. The PCR amplification was performed with cDNA as template and primers (Table 2) using SYBR^®^ Premix Ex Taq™ II (Tli RNaseH Plus), under the following conditions: 95°C for 30 s followed 40 cycles at 95°C for 15 s, 60°C for 40 s, and 72°C for 30 s. *hrdB* was amplified as the internal control. All cultures were analyzed by real-time PCR, with each specimen was copied in triplicate and RNA isolated from three independent cultures.

### Deposition of Nucleotide Sequence

The sequences reported in this study have been deposited in GenBank with accession no. MG871748.

## Results

### Sequence Analysis of the *wysPII* Gene

*Streptomyces albulus* CK-15 genome sequencing revealed that the *wysPII* gene product (WysPII; 952 amino acids, calculated molecular mass 101.82 kDa) showed 80.10% sequence identity with the *S. noursei* ATCC11455 protein NysRII (AF263912.1), which is a putative 953-amino acid regulatory protein encoded in the nystatin biosynthesis gene cluster. The putative protein sequence translated from the nucleotide was further analyzed using NCBI BLASTp showed 44.23% sequence identity of WysPII with another putative transcriptional regulator which has been reported in the tetramycin *ttmRII* biosynthesis gene cluster. WysPII has a HTH DNA-binding domain at its N-terminus, and its C-terminus is homologous to COG3899, which is predicted to be an ATPase. It also contains a MalT family domain, which regulates the *mal* regulon (Schlegel et al., 2002).

### Influence of *wysPII* Inactivation on Morphological Development and Antibiotic Production

**FIGURE 1:**
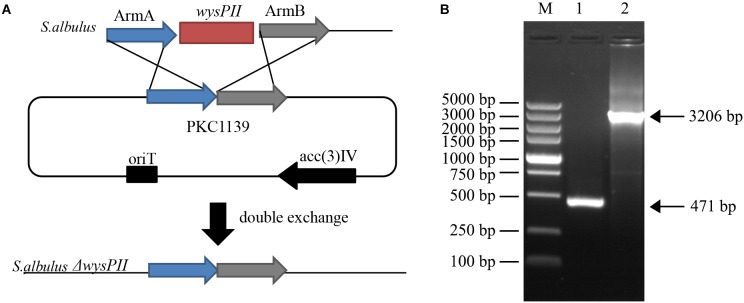
Identification of *wysPII* deletion **(A)** Gene replacement of *wysPII* in *Streptomyces albulus* CK-15. **(B)** Confirmation of the constructed Δ*wysPII* mutant by PCR. Lanes: M, BM 5,000-bp DNA ladder; 1, PCR verification with primers TF and TR using *S. albulus* Δ*wysPII* genomic DNA as the template; 2, PCR verification with primers TF and TR using *S. albulus* CK-15 genomic DNA as the template.

The roles of *wysPII* in wuyiencin production and morphological development were investigated in gene inactivation studies. Disruption vector pKC1139-Δ*wysPII* was transferred into *S. albulus* CK-15 by intergeneric conjugation and the *wysPII* gene was disrupted by homologous recombination (Figure 1A). pKC1139 is sensitive to temperature and replication is inhibited at temperatures >34°C; therefore, replication of the recombinant plasmid pKC1139-Δ*wysPII* in strain CK-15 was prevented in these conditions. Finally, a *wysPII* deletion mutant (*S. albulus* Δ*wysPII*) was selected by apramycin-resistance (Figure 1B).

**FIGURE 2:**
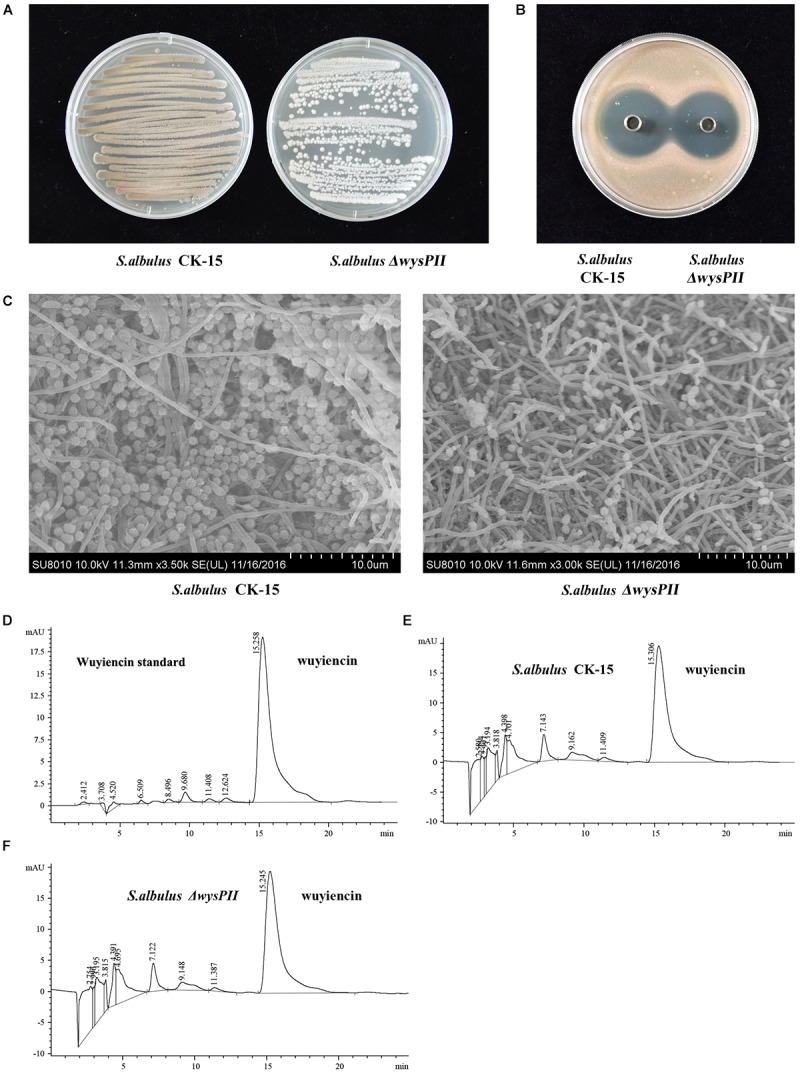
Effect of *wysPII* deletion on morphological development and wuyiencin production. **(A)** Phenotypes of 5-day-old *S. albulus* CK-15 and *S. albulus* Δ*wysPII*. **(B)** Antibacterial effect of *S. albulus* CK-15 and *S. albulus*Δ*wysPII* fermentation culture supernatant. **(C)** Electron microscopy of *S. albulus* CK-15 and *S. albulus* Δ*wysPII.* **(D)** HPLC analysis of standard wuyiencin. **(E)** HPLC analysis of wuyiencin production levels in *S. albulus* CK-15. **(F)** HPLC analysis of wuyiencin production levels in *S. albulus*Δ*wysPII.*

When grown on solid MS medium, the Δ*wysPII* mutant showed morphological changes that were apparent by both visual inspection and electron microscopy. Furthermore, the growth rate of the Δ*wysPII* was lower than that of the WT (Figures 2A,C). In addition, sporulation of ΔwysPII was reduced, and the ΔwysPII spores were much lighter in color than those of the WT strain. We also assessed production of wuyiencin in filtered fermentation broths of the WT and ΔwysPII strains. HPLC analysis revealed there were no differences in the wuyiencin production by the ΔwysPII and WT strains (1268.41 and 1280.93 mg/l, respectively; Figures 2D–F). Furthermore, crude extracts of the ΔwysPII broth were shown to inhibit *R. rubra* growth (Figure 2B). Thus, wysPII exerts a positive regulatory effect on the morphological development of *S. albulus* CK-15, but is not essential for wuyiencin biosynthesis.

### *Trans*-Complementation of the *wysPII* Mutant

**FIGURE 3:**
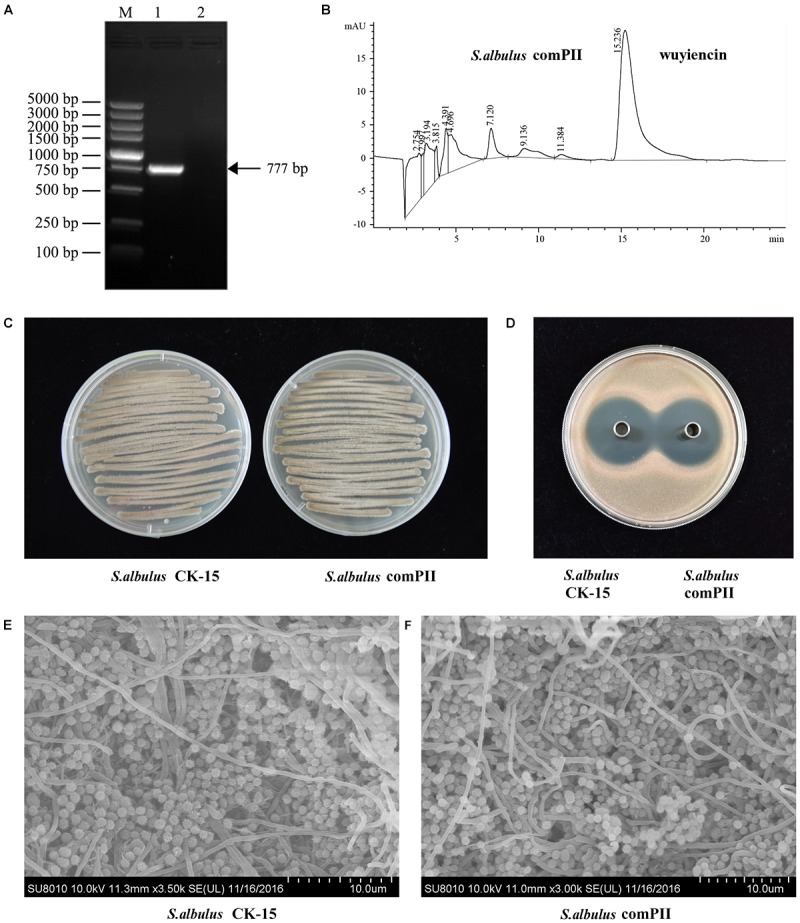
Effect of *wysPII* complementation on morphological development and wuyiencin production. **(A)** Confirmation of the complementation strain *S. albulus* comPII by PCR. Lanes: M, BM 5000-bp DNA ladder; 1, PCR verification with primers Am-F and Am-R using *S. albulus* comPII genomic DNA as the template; 2, PCR verification with primers Am-F and Am-R using *S. albulus* CK-15 genomic DNA as the template. **(B)** HPLC analysis of wuyiencin production levels in *S. albulus* comPII. **(C)** Phenotypes of 5-day-old *S. albulus* CK-15 and *S. albulus* comPII cultured on an MS plate. **(D)** Antibacterial effect of *S. albulus* CK-15 and *S. albulus* comPII fermentation culture supernatant. **(E)** Electron microscopy of *S. albulus* CK-15 and *S. albulus* comPII.

We also investigated *wysPII* in complementation studies of the deletion mutant. A DNA fragment containing *wysPII* and the putative promoter was inserted into integrative vector pSETC to generate pSETC-*wysPII*, which was then transferred from *E. coli* ET12567 (pUZ8002) to Δ*wysPII*. Apramycin-resistant transconjugants were confirmed by PCR amplification of a 777-bp fragment. The expected PCR product was observed only in the complementation strain *S. albulus* comPII (Figure 3A). Production of wuyiencin by the complementation strain was 1276.30 mg/L equal to that by the WT (Figures 3B,D), and no notable visual phenotypic differences were observed between the strains (Figures 3C,E). Thus, complementation of Δ*wysPII* with *wysPII* restored the WT phenotype in terms of morphological development.

### Effects of *wysPII* Overexpression on Morphological Development

**FIGURE 4:**
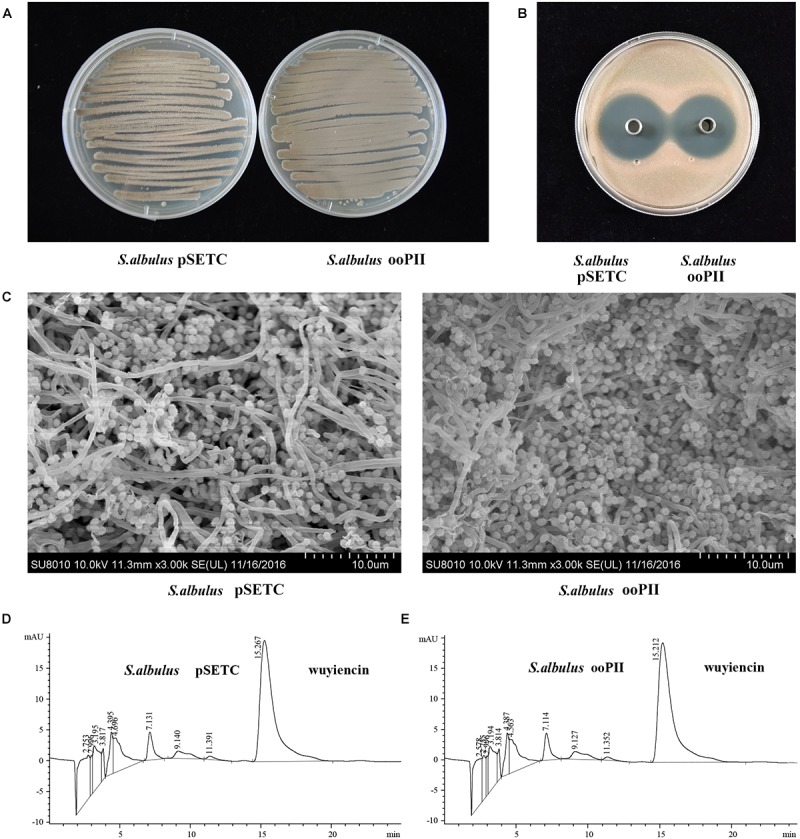
Effect of *wysPII* overexpression on morphological development and wuyiencin production. **(A)** Phenotypes of 5-day-old *S. albulus* pSETC and *S. albulus* ooPII. **(B)** Antibacterial effect of *S. albulus* pSETC and *S. albulus* ooPII fermentation culture supernatant. **(C)** Electron microscopy of *S. albulus* pSETC and *S. albulus* ooPII. **(D)** HPLC analysis of wuyiencin production levels in *S. albulus* pSETC. **(E)** HPLC analysis of wuyiencin production levels in *S. albulus* ooPII.

pSTEC-*wysPII*, with *wysPII* adjacent to promoter P_SF14_, was then transferred from *E. coli* ET12567 (pUZ8002) into *S. albulus* CK-15. The overexpression strain was designated *S. albulus* ooPII. As a positive control, the empty vector pSETC was also introduced into *S. albulus* CK-15 (*S. albulus* pSETC). Apramycin-resistant transconjugants were confirmed by PCR. There were marked differences in the levels of sporulation of the ooPII transconjugants and the WT strain. Accelerated growth was observed for the ooPII strain compared with that of the WT (Figures 4A,C). However, there were no differences in the inhibitory effects of *S. albulus* pSETC extracts on *R. rubra* growth compared with those mediated by the ooPII strain (Figure 4B), and wuyiencin production of *S. albulus* pSETC was 1278.90 mg/L unchanged compared with the overexpression strain (Figures 4D,E).

### The Wuyiencin Production Time-Course

**FIGURE 5:**
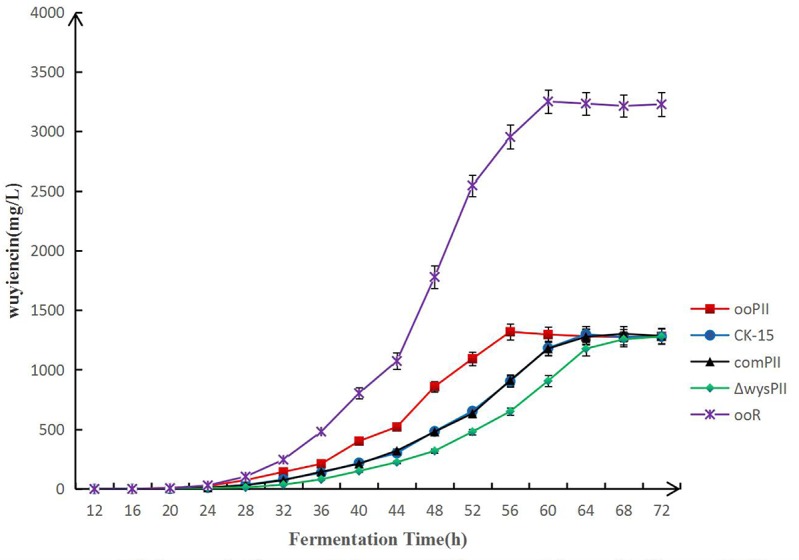
Time-course of wuyiencin production. Wuyiencin production by *S. albulus* CK-15, *S. albulus* ooPII, *S. albulus* Δ*wysPII, S. albulus* comPII and *S. albulus* ooR during fermentation for 72 h.

To compare the wuyiencin production time-course of the *wysPII* overexpression and WT strains, fermentation broth were collected at 2-h intervals by filtration using Millipore express membrane filters (pore diameter 0.22 μm; Merck) and analyzed by HPLC (Figure 5). The highest wuyiencin production by ooPII was detected after 56 h and 45.91% higher than that produced by the WT strain (1318.79 mg/L vs. 903.80 mg/L). The highest wuyiencin production by the WT strain was detected at 62 h (1307.53 mg/L). There was no significant difference in the greatest wuyiencin production between these two strains. Overexpression of *wysR*, which is a transcriptional activator of wuyiencin production, was also tested in the production time-course. The greatest wuyiencin production by ooR was observed at 60 h, which was almost threefold fasted than the time required by the WT strain.

### Effect of *wysPII* on Mycelial Biomass

**FIGURE 6:**
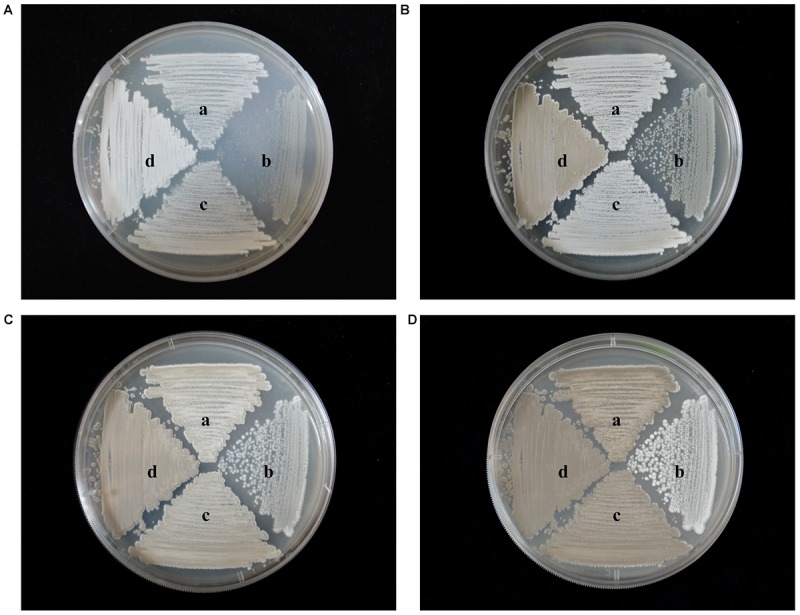
Morphological development of each strain on MS medium from 48 to 120 h. **(A)** The growth condition of different strains at 48 h. **(B)** Cultures of different strains after 72 h. **(C)** Cultures of different strains after 96 h. **(D)** Cultures of different strains after 120 h. (a) *S. albulus* CK-15, (b) *S. albulus* Δ*wysPII*, (c) *S. albulus* comPII, (d) *S. albulus* ooPII.

**FIGURE 7:**
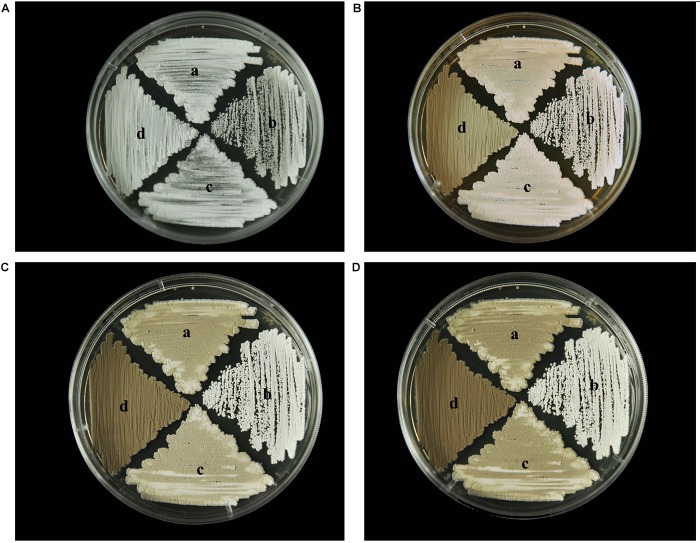
Morphological development of each strain on ISP2 medium from 48 to 120 h. **(A)** The growth of different strains at 48 h. **(B)** Cultures of different strains after 72 h. **(C)** Cultures of different strains after 96 h. **(D)** Cultures of different strains after 120 h. (a) *S. albulus* CK-15, (b) *S. albulus* Δ*wysPII*, (c) *S. albulus* comPII, (d) *S. albulus* ooPII.

**FIGURE 8:**
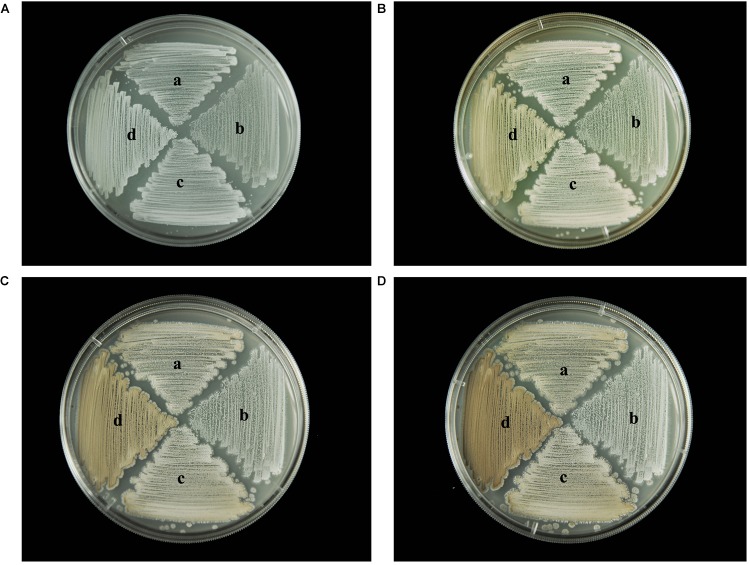
Morphological development of each strain on ISP3 medium from 48 to 120 h. **(A)** The growth of different strains at 48 h. **(B)** Cultures of different strains after 72 h. **(C)** Cultures of different strains after 96 h. **(D)** Cultures of different strains after 120 h. (a) *S. albulus* CK-15, (b) *S. albulus* Δ*wysPII*, (c) *S. albulus* comPII, (d) *S. albulus* ooPII.

**FIGURE 9:**
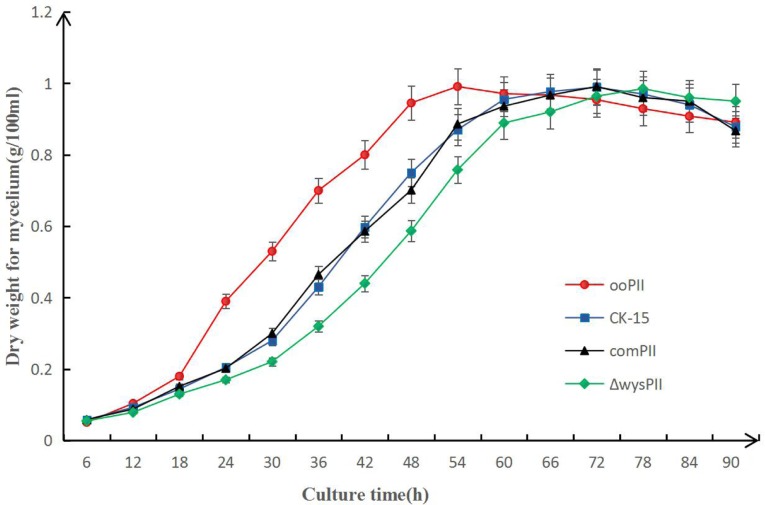
Result of mycelial biomass measurement. Biomass of *S. albulus* CK-15, *S. albulus* ooPII, *S. albulus* Δ*wysPII, S. albulus* comPII during culture for 90 h.

To identify any effects of *wysPII* on mycelial biomass, we studied the morphological development from 48 to 120 h of the WT. The Δ*wysPII*, comPII, and ooPII strains were cultured on MS, ISP2, and ISP3 solid medium, respectively (Figures 6–8). Spores were produced by ooPII at 48 h, whereas the WT and comPII strains had only reached the mycelial period of development at this timepoint, and the Δ*wysPII* strain had few mycelia. Gray spores were observed in the Δ*wysPII* strain by 72 h, while the spores of other three strains remained white. To monitor growth, samples were collected every 6 h and the mycelial biomass was determined (Figure 9). For the ooPII strain, the latent phase was from 0 to18 h, and the logarithmic phase was from 18 to 60 h. The greatest mycelial biomass was observed at 72 h for the WT, but ooPII showed more rapid growth, reaching the maximum biomass at 54 h. In contrast, Δ*wysPII* exhibited delayed growth, reaching its maximum at 78 h.

### Developmental Gene Expression Levels in *wysPII* Overexpression Strain

**FIGURE 10:**
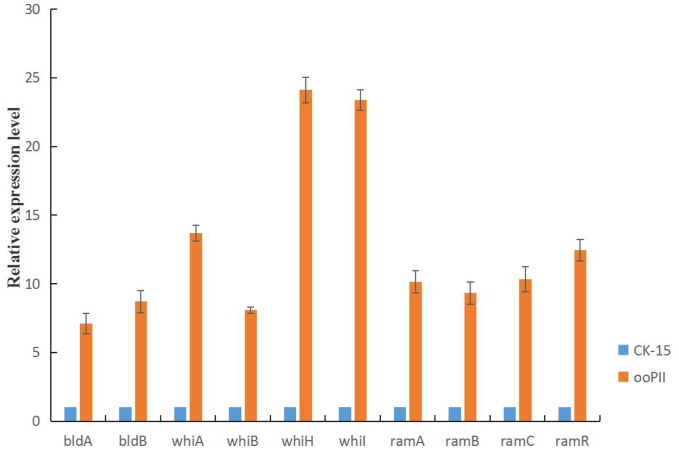
Developmental gene expression in *S. albulus* CK-15 and *S. albulus* ooPII by RT-qPCR. The RNA samples were isolated from 72-h cultures on MS medium. The relative values of *S. albulus* CK-15 are designated as 1. Data represent the mean ± standard deviation of three independent experiments.

To investigate the mechanism of the rapid growth and increased sporulation of the ooPII strain, we analyzed *wysPII* expression in the ooPII and WT strains by RT-qPCR. We also analyzed the expression of several genes of the *bld, whi*, and *ram* families, which are crucial for the morphological development of *Streptomyces*, especially for mycelial growth and sporulation (Keijser et al., 2000; Chater, 2001; Kim et al., 2014). All of the selected genes were highly overexpressed in the ooPII strain (Figure 10). The expression levels of seven of the genes (*whiA, whiH, whiI, ramA, ramB, ramC*, and *ramR*) were more than 10 times higher than those in the WT strain. Furthermore, it is worth noting that the expression levels of *whiH* and *whiI* in the ooPII strain were more than 20 times higher than those in the WT strain. These result suggested that *wysPII* activates these genes, which are crucial for the morphological development of *Streptomyces.*

## Discussion

Morphological development of *Streptomyces* has been studied mainly in *S. coelicolor* (Zhang et al., 2017). Although developmental regulation in *Streptomyces* is not completely understood, *bld* and *whi* genes have been shown to be involved (Flardh and Buttner, 2009). Mutations in *bld* block formation of aerial mycelia, resulting in a ‘bald’ appearance, whereas mutations in *whi* block steps in the conversion of aerial mycelia to mature, gray spores, and *whi* mutants appear white (Chater, 2011) because the mutants fail to produce the gray polyketide pigment that is visible in the mature WT spores (Den Hengst et al., 2010). In *S. coelicolor*, SapB, a small, hydrophobic, morphogenetic peptide, is crucial for differentiation into aerial hyphae and the *ram* gene cluster is indispensable for SapB production (Kodani et al., 2004). In this study, the *wysPII* mutant exhibited slower growth and delayed sporulation compared with WT *S. wuyiensis* CK-15. In addition, the Δ*wysPII* strain produced white spores, while those produced by the WT strain were gray. The Δ*wysPII* strain showed the same phenotypic features as mutants of the *whi* family, which is inconsistent with the effects of mutations reported in other LuxR family regulators. All the selected genes (*bldA, bldB, whiA, whiB, whiH, whiI, ramA, ramB, ramC*, and *ramR*) were highly overexpressed in the ooPII strain (Figure 10), especially *whiH* and *whiI.* This result suggests that *wysPII* activates these genes, which are crucial for the morphological development of *Streptomyces.* Furthermore, the ooPII strain grew much faster than the WT strain and exhibited increased sporulation. Most LuxR family regulators have been reported as activators of antibiotic production in *Streptomyces* (Yu et al., 2012). *S. natalensis* strains expressing a mutant form of PimR, which is a putative regulator of pimaricin production, exhibited the same growth and morphological characteristics as the WT strain (Antón et al., 2004). WysR is a luxR family transcriptional activator of wuyiencin biosynthesis and morphological development (Liu et al., 2014). Very few LuxR family members have been reported to regulate the morphological development of bacteria (Vicente et al., 2014). In a study of positive regulators of tetramycin biosynthesis in *S. ahygroscopicus*, colonies of *ttmRII* mutants showed less gray pigmentation and no regulatory effect on antibiotic production was observed (Cui et al., 2016).

WysPII is a multidomain protein, which was found to contain a MalT family regulator domain, with the C-terminal HTH motif that is a positive activator of transcription of the *mal* regulon in *E. coli* K-12 (Panagiotidis et al., 1998). Mutants without *malT* are unable to grow on maltose, and strains with mutations in this domain show constitutive expression of the *mal* gene (Schlegel et al., 2002). However, the WT, ooPII and Δ*wysPII* strains exhibited growth retardation and reduced sporulation when cultured with maltose as the only carbon source. Thus, the *malT* may have no obvious effect on the morphological development of *S. albulus* CK-15.

The results of the present study indicate that *wysPII* functions as an activator of morphological development, with *wysPII* overexpression leading to more rapid growth than that of the WT and *wysPII* deletion leading to retardation of morphological development. In the *wysPII* overexpressing strain, large quantities of spores were produced by 72 h when cultured on solid MS medium, while spore production by the WT strain was only observed after 96 h (Figure 6). In liquid culture, the *wysPII* overexpressing strain grew faster and reached maximum biomass at 54 h, approximately 18 h faster than the WT (Figure 9). In terms of wuyiencin production, the maximum levels were generated by the ooPII strain at 56 h, while this occurred at 62 h in the WT strain (Figure 5). Improving the efficiency and reducing the cost of wuyiencin production is essential to promote of its use as an agricultural biopesticide to control plant fungal diseases; the use of overexpressing strains may be advantageous in industrial production processes.

## Author Contributions

KZ conceived and supervised the studies. BL and BG performed all experiments and wrote the paper. JM, QW, AK, and LS analyzed the data.

## Conflict of Interest Statement

The authors declare that the research was conducted in the absence of any commercial or financial relationships that could be construed as a potential conflict of interest.
